# Personality Traits and Depression in Infertile Couples during the COVID-19

**DOI:** 10.3390/jcm13164827

**Published:** 2024-08-15

**Authors:** Sofia Burgio, Concetta Polizzi, Marianna Alesi, Antonio Perino, Gaspare Cucinella, Yulia Kotlik, Alessandra Lopez, Alessandra Vassiliadis, Giuseppe Gullo

**Affiliations:** 1 Department of Obstetrics and Gynaecology, IVF UNIT, Villa Sofia—V. Cervello Hospital, University of Palermo, 90128 Palermo, Italy; 2 Department of Psychology, Educational Science and Human Movement, University of Palermo, 90128 Palermo, Italy; 3 Department of Health Promotion, Mother and Child Care, Intermal Medicine and Medical Specialties, University of Palermo, 90128 Palermo, Italy

**Keywords:** infertility, personality traits, depression, COVID-19, assisted reproductive technology (ART)

## Abstract

**Background/Objectives**: The study presented in this paper seeks to examine how personality traits and depressive symptoms, influenced by the fear of COVID-19, interact in infertile couples, who are on the verge of commencing treatments with assisted reproductive technology (ART). The purpose of this observational study was to explore the relationship between personality traits in infertile couples and the occurrence of depressive symptoms, taking into account the mediating effect of COVID-19 fear. **Methods**: The study sample consisted of 108 women and 71 men (N = 179), all of whom had received an infertility diagnosis and expressed a desire to begin ART treatment at a Sicilian ART center; they were subsequently recruited. The Personality Inventory (PI), Beck Depression Inventory (BDI) and Fear of COVID (FCV-19S) were used for data collection. **Results**: The FCV-19S demonstrates a significant positive correlation with both neuroticism (r = 0.25, *p* = 0.001) and agreeableness (r = 0.19, *p* = 0.012). In addition, there is a significant correlation between FCV-19S (r = 0.67, *p* < 0.001) and depression symptoms. The mediation analysis reveals that neuroticism is a predictor of fear of COVID-19 (β = 1.77, *p* = 0.001) and depression (β = 0.22, *p* = 0.002). Additionally, the fear of COVID-19 significantly influences (β = 0.12, *p* < 0.001) depression. **Conclusions**: This study found that neuroticism and agreeableness are positively linked to the fear of COVID-19, and women displayed notable mild mood disorders. Neuroticism predicted both depression and fear of COVID-19, while fear of COVID-19 predicted depressive symptoms. However, the total efficacy of the mediation model was not significant, thereby suggesting that the variables do not fully explain this model.

## 1. Introduction

Many consider that assisted reproduction technology (ART) treatments represent the most significant scientific advancement in the search for solutions to address infertility and the difficulties of spontaneous conception in achieving desired parenthood. Previously considered as uncommon and atypical conditions, these issues are now increasingly prevalent due to the advancing age of mothers [1] and various environmental factors.

It is worth noting that, from 2013 to 2021, there was an exponential increase in the number of couples undergoing first, second, and third level ART techniques (from 71,741 couples in 2013 to 86,090 in 2021), and that Sicily has a high percentage of currently active ART centers [2] (Activities of the Italian National ART Registry—2021).

Infertility is identified as the inability to achieve pregnancy after one year of attempting through frequent and unprotected sexual intercourse, or as the inability of an individual or couple to conceive [3]. This condition, which affects 10 to 15% of couples worldwide, with half of the cases attributed to the male factor [4], represents a significant public health issue, both in terms of increasingly concerning low birth rates and costs [3,5]. The number of couples in Italy experiencing difficulties in natural conception has doubled since 1996, reaching a proportion of one in five [6].

From a psychological perspective, infertility is particularly burdensome for both men and women. It deeply affects individual and couple well-being [7]. Symptoms related to depression and anxiety are the most frequent reactions to infertility and the beginning of an ART journey [8]. The high prevalence of depressive and/or anxiety disorders raises the possibility that certain personality traits may predispose individuals to the development of depression and anxiety in response to the condition of infertility, in addition to the emotional burden of treatments to achieve a pregnancy [9].

A longitudinal study conducted in Italy has highlighted that 17.9% of the women involved in such treatments exhibited depressive symptoms, while 14.7% displayed symptoms of anxiety. However, depressive symptoms affected 6.9% of men and symptoms of anxiety affected 4.5% of cases [10]. Depression, compared to anxiety, therefore, seems to be more prevalent among infertile couples. Furthermore, a survey of 43 women has revealed that certain beliefs regarding motherhood and the desire for ‘perfectionism’, perceived as a personality trait, are associated with lower levels of fertility-related quality of life [11].

According to the Big Five theory of personality traits, Costa and McCrae [12] have proposed the following taxonomy, which is based on five major factors: extraversion, neuroticism, agreeableness, conscientiousness, and openness to experience. Various studies have demonstrated that there are personality differences in determining reproductive behavior [13,14].

Neuroticism reflects a general tendency to experience negative emotions, such as anxiety, and to become easily distressed. Neuroticism has been associated with difficulties in social relationships, such as lower relational quality and interpersonal negativity in marriage [15,16]. Furthermore, neuroticism has been linked to intentions of procreation. In a sample of young German adults, individuals with high neuroticism displayed greater decisional ambivalence regarding the idea of becoming a parent [17]. Such decisional ambivalence can lead to postponing parenthood, in addition to having fewer children [14] and, therefore, to seeking ART, also due to advanced maternal age.

In light of this review of studies in the literature, it is of great interest to understand which relationships might exist between such personality traits and depressive symptoms, in a social context mediated by the presence of the COVID-19 pandemic.

In the context of the latter [18], the main scientific and professional societies regarding the field of fertility [19,20] have advised postponing a pregnancy [21] insofar as monitoring an ART journey requires continuous checks, which are not only gynecological but also hormonal. With a rationale of containing the pandemic emergency, with measures such as social distancing and lockdown, it was preferable to advise delaying taking all unnecessary risks. Moreover, ART services are often located in facilities not immediately adapted to respecting safety guidelines issued by governments around the world in containing the pandemic.

Personality traits can have a significant impact on how individuals perceive threats and react to crises like pandemics [22]. Individuals with tendencies towards neuroticism, anxiety, emotional instability, and lack of security have shown an increase in stress levels and negative emotional reactions [23]. Caci and colleagues [24] observed that individuals with a high degree of neuroticism exhibited greater fears related to the effects of COVID-19 in everyday life, tending to experience boredom more easily and to indulge in negative thoughts and fantasies.

Precisely because neuroticism is identified, according to the Big Five model [12], as the primary trait influencing the management of fear, people with a high level of neurotic traits may be more susceptible to perceiving danger, an intense emotional response, and pay greater attention to threats, with a reduced capacity to deal with stressful situations [25]. Although the stress related to fertility can be considered comparable to that generated by the pandemic [26,27], the emotional impact of the pandemic on infertile patients is significant [28].

In light of what has been presented, the purpose of this observational study is to investigate personality factors in infertile couples and their relationship with the presence of any depressive symptoms, considering the role of fear of COVID-19 as a mediating variable.

This study, therefore, has the following hypotheses:

– To verify the existence of significant correlations between personality factors, depression, and fear of COVID;
– To verify the relationship between personality traits and depression, taking into account the role that a fear of COVID-19 may have played in mediating such an association, in infertile men and women who were about to commence a medically assisted reproduction (MAR) journey.

## 2. Materials and Methods

### 2.1. Participants

A total of 108 women and 71 men (N = 179), who had received an infertility diagnosis and expressed a desire to start ART treatment at a Sicilian ART center, were recruited. The study was conducted over the course of one year from July 2021 to July 2022, and the inclusion criteria were to have received an infertility diagnosis and an understanding of Italian for the administration of psychometric tests. Some cases were excluded where questionnaires were incomplete.

The sample characteristics (see Table 1) confirmed that the study participants were mostly couples from a middle–low socioeconomic and cultural background. Indeed, the assisted reproduction center, where the participants were recruited, is located in an area particularly at risk of socioeconomic disadvantage. The center, therefore, serves as the only clinic offering ART services at reduced costs compared to other centers.

### 2.2. Procedures and Instruments

The survey sample was recruited during the first gynecological visit, following a clinical interview in which couples were invited to participate in the research, and where informed consent to the study was provided and read. Participation was voluntary and anonymous, and the administration time of the psychometric tools lasted for approximately 20 min.

The research journey included the use of the following measures:

– A socio-demographic form, constructed ad hoc, in which the following data were collected: sex, age, nationality, relationship status, educational level, profession type (where relevant), number of children, incidence of previous miscarriages, information relating to whether it was the first attempt at ART, presence of previous pathologies, healthy eating habits, engaging in physical and sports activities, smoking habit, alcohol consumption, use of pharmacotherapy, and the presence of a social support network.
– Personality Inventory (PI) [29], a self-reporting questionnaire consisting of 20 items to assess personality factors, according to the Big Five model [12]. The questionnaire has five subscales which investigate the relative personality factors: extraversion, defined as a search for aggregation, assertiveness, positive emotionality, and a search for excitement; conscientiousness, defined as a sense of duty and self-discipline; openness regarding experiences and intellectual curiosity; agreeableness, defined as trusting in others and the ability to cooperate; and neuroticism, defined as a tendency to be emotionally unstable. Each item had a 5-point Likert scale with scores ranging from 1 = strongly disagree to 5 = strongly agree. Regarding psychometric properties, the tool demonstrated reliable internal consistency (α > 0.70).
– Beck Depression Inventory II (BDI) [30] is a self-reporting questionnaire consisting of 21 items; its aim is to measure the cognitive, motivational, affective, and behavioral symptoms of depression. Each item has a score ranging from 0 to 3, and the higher the BDI score, the more profound the degree of depression is. Regarding psychometric properties, the tool demonstrated reliable internal consistency (α > 0.80).
– A fear of COVID-19 (FCV-19S) [31] is a seven-item scale which assesses the fear of COVID-19. The seven items are rated on a 5-point Likert scale from 1 (strongly disagree) to 5 (strongly agree), with scores ranging from 7 to 35. The higher the score, the greater the fear of COVID-19. Regarding psychometric properties, the FCV-19S also demonstrated reliable internal consistency (α > 0.80).

### 2.3. Statistical Analyses

Preliminary analyses (means, SDs, and percentages) were calculated for the various socio-demographic variables. The variables being studied were investigated by performing Pearson correlations among personality factors, depression, and fear of COVID-19. An independent samples *t*-test was conducted to investigate the difference between the mean values of men and women for the depression variable. Finally, a mediation model was constructed in order to investigate the relationship between personality traits and depression, exploring the mediating role of the COVID-19 fear variable. Confidence intervals were reported, and bootstrapping was used to estimate these intervals. The significance of the indirect effect is indicated if the interval between the upper and lower confidence limits does not contain zero [32]. All analyses were performed using the SPSS statistical software version 28 (IBM SPSS Statistics).

## 3. Results

The results demonstrated that the average age of the women in the study was 34.6 (SD = 5.65), while that for men was 37.3 (SD = 5.93). The majority of women (96.3%) and men (97.2%) were Italian. The most prevalent relationship status was ‘marriage’ for women (75.9%) and men (77.5%). Regarding education level, 35.3% of women and 31% of men had completed lower secondary education. Approximately 38.3% of the women were housewives, while the majority of men were employed (34.3%). Additionally, 85% of the women did not already have children, similar to the majority of men (85.5%).

In total, 72.9% of the women reported having had no previous miscarriages, in addition to not having any psychological or organic pathological conditions apart from infertility; this statement was shared by 71.3% of the women and the majority of men reported the same characteristic (84.6%).

Although most women (83.3%) and men (78.3%) claimed to follow a healthy diet, the majority of them did not engage in any sports or physical activity (77.8% of women and 59.4% of men). Similarly, a regular smoking habit (70.4%), alcohol consumption (95.4%), and medication (77.8%) were absent among women. Men displayed similar habits, with 59.4% stating that they were non-smokers, 94.2% confirming they did not regularly consume alcohol, and 81.2% did not regularly use medication.

While the majority of women described their social network as “limited” (54.2%), most men referred to having an extensive social network (57.4%). Finally, both women (62.9%) and men (53%) reported having their family as the main reference point regarding support and emotional backing in starting their ART journey.

Descriptive statistics are reported in Table 1 above.

The results of the correlations are shown in Table 2 below.

Additionally, the independent samples *t*-test indicated a difference regarding the depression variable [t (1177) = 15.75, *p* < 0.001].

Descriptive analyses highlighted BDI scores greater than 10, that is, the cut-off point for identifying a mild mood disorder in women (M = 12.8; SD = 1.6) but not in men (M = 4.6; SD = 5).

Based on the results obtained from the correlations, and from the literature, mediation models were developed in which the relationships between personality factors, fear of COVID-19, and depressive symptoms were analyzed.

The mediation hypothesis was examined by testing the significance of the indirect effect of fear of COVID-19 on depressive symptoms.

**Figure 1:**
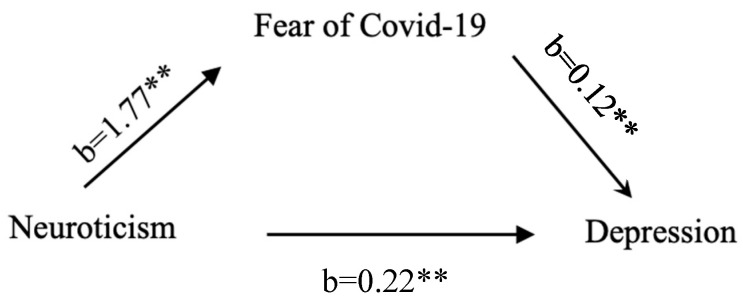
Mediation model.

The results of the mediation analyses performed with Hayes’ procedure [33] are presented in Figure 1 below.

The results demonstrated that neuroticism predicted a fear of COVID-19 (β = 1.77, ** *p* = 0.001), as well as depression (β = 0.22, ** *p* = 0.002). The mediator, fear of COVID-19, also significantly predicted (β = 0.12, ** *p* < 0.001) the depression variable. In conclusion, the mediation test indicated that neuroticism directly influenced depression (*p* = 0.002), and the effect of the mediator, fear of COVID-19, was also significant (SE = 0.016, 95% CI = [0.02, 0.08]). However, no total effect of the model was found to be significant (*p* = 0.96).

## 4. Discussion

The results of the observational study have demonstrated that a fear of COVID-19 is significantly correlated with neuroticism and the personality trait of agreeableness. The correlation between fear of COVID-19 and neuroticism aligns with the literature in the field, which indicates that this personality trait is more closely associated with the presence of anxiety disorders, depression, and generally a lower level of satisfaction with one’s quality of life [34,35,36]. Other studies that have examined different variables and sample characteristics have reported strong associations between neuroticism and various mental health problems, thereby highlighting how this personality trait is a significant risk factor, even in non-pregnant populations [37]. Indeed, as is known, personality traits are predictors of significant health outcomes as they also affect physical illnesses and influence an individual’s mental health [38]. Thus, as supported by other studies in the literature, having a personality with high levels of neuroticism increases the fear of COVID-19 [39], and this was also detected in a predictive sense. On the other hand, the correlation between fear of COVID-19 and agreeableness is inconsistent with studies in the literature [40,41], which suggests that this personality trait corresponds to a greater acceptance of the changes imposed by COVID-19, in addition to the presence of less dysfunctional behaviors.

It could be hypothesized that, in the sample examined, being willing and open to meeting others (a characteristic typical of individuals who report high levels of agreeableness) may increase the perception of a greater fear of social contact, which is so central in the organization of their personality. Furthermore, results from other recent studies have suggested that individuals with high levels of agreeableness (in particular) have tended to comply more with government rules and recommendations to combat COVID-19 [42]. These data may also be supported by the correlation between agreeableness and a fear of COVID-19.

A significant finding supported by studies in the literature is the correlation between fear of COVID-19 and depression [43,44,45]. Indeed, many research paths have supported the study’s results. Indeed, a fear of COVID-19 has been positively correlated with mental health consequences such as depression, stress, and anxiety symptoms, indicating that the higher the FCV-19S score, the higher the level of mental health issues such as depression [46]. Çıkrıkçı et al. [47] have shown that the fear of illness and infection can lead to depression, anxiety, and stress in some people. In a similar vein, Huang and Zhao [48] have observed that many individuals experienced moderate-to-severe levels of depression, anxiety, and stress during the initial phase of the COVID-19 pandemic. Yet, another study described the positive relationship between anxiety, stress, depression, intolerance to uncertainty, FCV-19S, and positivity, finding mediators. This research indicated a statistically significant positive correlation between FCV-19S and depression, intolerance to uncertainty, anxiety, and stress [49].

Equally significant is the difference between the mean scores of men and women regarding the scores reported on the BDI; women displayed higher scores, indicating, according to the scoring provided by the psychometric tool, the presence of a mild mood disorder. This finding is also supported by already published studies which emphasize how depression is the condition most widespread among infertile women [50], compared to men who report lower scores [51,52].

There are many studies in the literature regarding degrees of depression because depression is a widespread condition and it is often related to infertility [50,53]. At present, depression is the most frequently diagnosed psychiatric condition worldwide [54,55]. Moreover, even after treatments with assisted reproduction techniques, couples sometimes fail to overcome the feelings of mourning and loss associated with infertility [56]. Additionally, it is important to highlight that infertility also contributes to the risk of depression. According to Nik Hazlina et al. [57], women experiencing infertility are 1.4 times more likely to suffer from depression. This indicates that infertility elevates the risk of depression. Therefore, this finding should be considered a guiding factor for clinical and research interventions.

Of greater significance is the average score reported by women on the BDI (>10), which highlights the presence of a mild mood disorder, suggesting clinical indications regarding the intervention to be made with these women. Indeed, even regarding couples undergoing their first attempt of ART, health professionals should bear in mind these clinically significant data and particularly consider the emotional aspect of the infertility condition, often omitted in the gynecology department [58,59].

Moreover, the mediation analysis revealed how neuroticism predicted a fear of COVID-19, as well as having a significant effect on depression. Neuroticism is a personality trait which was originally defined to include anxiety, emotional instability, worry, tension, and self-pity. This negative affectivity can be accompanied by a pervasive perception that the world is a dangerous and threatening place, along with beliefs about one’s inability to manage or cope with difficult events [60]. In agreement with previous studies [24,61,62], the results confirmed Taylor’s [63] prediction that individuals with a high level of neuroticism are vulnerable to significant discomfort during pandemics because they are sensitive to stress and infection threats. Thus, having a personality with high levels of neuroticism can predict a real fear of COVID-19.

The role of neuroticism as a predictor of depression is also supported by the literature in this field [64,65]. Such studies suggest, for example, a relationship mediated by the environment between neuroticism and depression. This personality factor interacts with stressful life events, triggering new episodes of depression at young and more advanced ages [66,67]. Similarly, the pandemic can be understood in this sense as a definitively stressful life event, which, in addition to predicting the depression variable, permits the results obtained from the mediation model within the considerations already made by studies in the literature. A note of caution is that although these direct and indirect relationships in the mediation model are significant (with some aligning with the existing literature), the fact that the total effect of the mediation model is not significant suggests the need to consider other variables not examined in this study in order to explain the relationship between neuroticism and depression, with a fear of COVID-19 as a mediator.

## 5. Conclusions

The study has highlighted how the well-being of infertile couples can be significantly compromised by certain personality traits and the potential presence of depression, especially during global emergencies such as the COVID-19 pandemic. Some correlations, such as the relationship between neuroticism and a fear of COVID-19, are supported by the current literature. Other innovative findings have also been highlighted in this study; for example, the correlation between agreeableness and a fear of COVID-19 diverge from the current literature in the field.

The presence of mild mood disorders in the average scores of infertile women underscores the importance of addressing the well-being of these patients from their initial consultation regarding ART services. This suggests a shift in clinical practice which should focus not only on addressing infertility itself but also on empowering infertile women psychologically by leveraging their resources. In addition, and as highlighted by the mediation model, certain personality traits, such as neuroticism, are effective predictors of the fear of COVID-19 and the presence of depressive symptoms. Similarly, the authors of this study noted that perceiving an intense fear of COVID-19 predicted significant depression. However, while these individual relationships are significant, the total impact of the model cannot be said to be significant, indicating that the mediation model is not fully explained by the variables examined. This suggests that the study may have required the elaboration of additional variables.

The consequences of this study may have an impact on various fields, including clinical practice, future research, and the understanding of psychological dynamics in healthcare emergencies. The finding that neuroticism is a strong predictor of depression and a fear of COVID-19 highlights the importance of assessing personality traits in the psychological evaluations of patients. Mental health professionals may find it efficacious to implement more targeted screenings and personalized interventions in addressing symptoms of depression and anxiety, especially in emergency contexts such as pandemics. The presence of mild mood disorders among women suggests the need for additional psychological support, even during the initial consultation with assisted reproduction services. Specialists in the field might consider integrating psychological interventions into treatment protocols to improve the overall well-being of patients.

Finally, the insignificance of the overall effect of the mediation model suggests that other variables might influence the relationship between neuroticism, a fear of COVID-19, and depression. Future research could explore additional confounding or mediating factors, such as social support, resilience, or past trauma experiences. Since the mediation model did not demonstrate a significant effect on the mental health of patients, longitudinal studies tracking participants over time could provide further insights into how these relationships evolve and impact on mental health.

In light of the considerations presented in this paper, infertility in couples can be said to be a heterogeneous condition, with great variability. Studying the personalities of men and women who access ART services, and the potential impact of depression, are undoubtedly variables of primary importance when dealing with infertility. This is particularly relevant if studies can promptly increase the quantity and quality of evidence-based clinical interventions. Of the limitations of this study, the lack of a variable with which to measure anxiety should certainly be highlighted; such an inclusion may have clarified the relationship between neuroticism and depression.

## Figures and Tables

**Table 1:** Descriptive characteristics of the sample (N = 179).

Variables	Women		Men	
	Mean	SD	Mean	SD
Age:	34.6	5.65	37.3	5.93
Nationality:				
Italian	75.9%		77.5%	
Other	24.1%		22.5%	
	96.3%		97.2%	
	3.7%		2.8%	
Relationship status:				
Married	75.9%		77.5%	
Cohabiting	24.1%		22.5%	
Educational level:				
Elementary school	5.6%		1.4%	
Middle school	35.1%		31%	
Vocational diploma	9.3%		25.4%	
High school diploma	24.1%		28.2%	
Bachelor’s degree	21.3%		11.3%	
PhD/Specialization	4.6%		2.8%	
Job type:				
Homemaker	39.2%		1.4%	
Student	2.8%			
Factory worker	0.9%		18.6%	
Office worker	32.7%		34.3%	
Executive	2.8%		1.4%	
Craftsman/woman			7.1%	
Merchant	0.9%		2.9%	
Freelancer	0.9%		17.1%	
Teacher	4.7%		1.4%	
Unemployed	10.3%		15.7%	
Seeking initial employment	4.7%			
Presence of children:				
No	85%		85.5%	
1	14%		13%	
2			1.4%	
3 or >3	0.9%			
Miscarriages:				
No	72.9%			
Yes, spontaneous	24.3%			
Yes, voluntary termination of pregnancy	2.8%			
First attempt at ART:				
Yes	72.9%		69.5%	
No	27.1%		30.5%	
Presence of other Pathologies:				
No	71.3%		84.6%	
Yes, organic type	23.1%		12.3%	
Yes, psychological type	0.9%			
Healthy carrier	4.6%		3.1%	
Healthy diet:				
Yes	83.3%		78.3%	
No	16.7%		21.7%	
Sport:				
Yes	22.2%		40.6%	
No	77.8%		59.4%	
Smoking:				
Yes	29.6%		40.6%	
No	70.4%		59.4%	
Alcohol:				
Yes	4.6%		5.8%	
No	95.4%		94.2%	
Taking medication:				
Yes	22.2%		18.8%	
No	77.8%		81.2%	
Social support network:				
Limited	54.2%		42.6%	
Wide	45.8%		57.4%	
Support system for ART journey:				
Family	62.9%		53%	
Friends	3.7%		4.5%	
Both	33.3%		42.4%	

Fear of COVID-19 is significantly and positively correlated with neuroticism (r = 0.25, *p* = 0.001), as well as with agreeableness (r = 0.19, *p* = 0.012). Furthermore, fear of COVID-19 (r = 0.67, *p* < 0.001) is significantly correlated with depressive symptoms.

**Table 2:** Correlations between personality factors, depression (BDI), and fear of COVID-19 (FCV-19S) in infertile couples.

Variables	1	2	3	4	5	6	7
1. Neuroticism	-						
2. Conscientiousness	−0.071	-					
3. Openness	−0.051	0.107	-				
4. Extroversion	−0.035	0.059	0.152	-			
5. Agreeableness	0.102	0.035	0.021	−0.011	-		
6. BDI	−0.004	−0.009	0.043	0.016	−0.004	-	
7. FCV-19S	0.252 **	0.050	0.141	0.072	0.188 *	0.666 **	-
M	8.78	15.06	12.4	12.9	13.1	9.63	13.7
SD	3.07	2.23	2.46	1.80	2.40	5.27	6.41
Skewness	0.91	−0.81	0.23	0.20	−0.14	−0.66	1.13
Kurtosis	1.09	1.55	0.37	1.9	0.38	−0.60	1.07

* *p* < 0.05; ** *p* < 0.01.

## Data Availability

The data presented in this study are available on request from the corresponding author.
